# Epistolary

**Published:** 1871-05

**Authors:** 

**Affiliations:** Chicago


### Epistolary.

My Dear Professor:

We have just returned home physically exhausted, having been engaged in a gigantic struggle to wheedle, or persuade several of our patrons to shell out, and give us something more substantial than air to live on; but, as they remain obdurate, and can't see it in our light, we are obliged to accept the situation, and hence refresh ourselves by reading the discussion on metro-peritonitis and opium, as published in the last two numbers of the Journal. As we feel in a proper mood to do almost anything desperate, and as we do not feel particularly interested in the controversy, we adopt the rôle of most intermeddlers, under like circumstances, and pitch in; firstly, because our mental irritation is such, that a literary stimulus is necessary; and, secondly, because, in this glorious, and enlightened free republican government of the U. S. A., fair play is a jewel. And we don't like to see two on one. So, figuratively speaking, we take off our coat, roll up our sleeves, put on the gloves, and call time.

First Round. *We do not believe the case one of metro-peritonitis, and hence a false diagnosis.*

A celebrated accoucheur and physician in England (Clark), many years ago, established a rule that, no puerperal woman was to be considered safe, so long as the pulse kept up to 100; not that disease actually existed, but that it was a guide to keep our attention alive to the probabilities. In this patient, the symptoms were entirely too inconstant and disconnected, too vague and ephemeral, for us to build a certain diagnosis upon; true, subjectively there was great pain and tenderness upon pressure, but the rational symptoms were far too mild, and disappeared too suddenly, even if the area of inflammation, supposing that any did exist, was confined below the superior strait. The pulse but once, the second day, ran up to 110, falling the next to 100 or less, and never again rising above that standard. The condition of the circulation is the most certain guide, not only to treatment, but of the pathological condition of the patient, and we affirm that, very few cases of peritoneal inflammation are treated with a pulse less than 120, but much oftener 140, or even 160, *never* full and bounding, *always* hard and jerking. There are not many obstetrists but have witnessed within the first three or four days after labor, cases of high nervous excitation, attended by pain and tenderness, abdominal distention, deficient secretions, etc., etc., so alarming at the time, especially if connected with perversions of intellect, as to give the greatest anxiety, and lead to vigorous measures for their suppression; but, no matter what the treatment, provided it be not heroic, as the condition is transitory, they soon subside,—the milk begins to secrete, and in a few days safety is secured.

In the case under consideration, the trouble began on the 20th with very severe symptoms, but on the 22nd inst., at 2 o'clock in the morning, having slept from 11, just *three* hours,—and how she got even this much rest, with two or three grains of opium popped into her every half hour, the Lord only knows,—she awakened *bright*, *intelligent*, and *feeling well*, and would have convalesced from that moment, only 44 hours ill, had not the stupid nurse, unmindful of the attending physician's directions, given, at intervals, *six* grains more, which produced immediate narcosis, though she had already within her 123 grains. But just here, our doctor fortunately recognized the power of the six grains, and with consternation in his face, and coat tails at right angles to the small of his back, fled incontinently to seek guidance and advice from his *confrères*,—a thing he had done several times before under similar cases of opium-poisoning—or rather when "the indications were more than met,"—and by continuous injections of atropine hypodermically, etc., etc., brought his patient through, or, to use his own expression, the alarming symptoms

"quickly disappeared with appropriate treatment," *i. e.*, by the very antagonist of opium.

Right duke well thrown up from the hip, alighting square between his blinkers; claret flowed briskly; and went to grass. First blood, and knock down for us.

SECOND ROUND. *Not being a case of metro-peritonitis the amount of opium claimed was never given, the patient being now alive.*

We all know, that in severe inflammatory diseases of serous membranes, especially if the uterine tissues be involved, the nervous system becomes so obtunded,—and we use this word as not incompatible with hyper-irritation,—that secretion, digestion, absorption, etc., are almost completely arrested, and that large doses of powerful drugs may be given without producing their full physiological effects, not because the nervous system cannot be awakened to their influence wholly, but mostly because they cannot be absorbed into the circulation. Many authors tell us that, in such diseases as tetanus, hydrophobia, chorea, etc., where formerly large doses of opium were administered in the solid or crude form, the microscopic revelations showed quantities of this drug in the stomach (and bowels), undigested and almost untouched, and hence recommended it to be given in the fluid state, as much more likely to reach the nervous centers. Of course it is astonishing how much opium may be forced upon an unfortunate, but the astonishment will very materially lessen, when we feel assured that the larger portion will remain as inert as just so much putty, and to prove that this tolerance arises in the main from this physiological fact, we have only to inject the ordinary doses into the venous circulation, to acknowledge how promptly it will produce its effects, and how dangerously too, if we dare push the remedy to extremes. We were an *interne* in Bellevue Hospital about the time that great and justly celebrated medical philosopher, Prof. A. Clark, was carrying out his treatment in peritoneal disorders, and had several cases under observation in the lying-in ward of our senior and others, but the patients died notwithstanding, and no more conclusive evidence of its failure can be adduced, than the simple fact that, though recommended by such high authority, it has been generally discarded by the profession, and, if we mistake not, is not now employed in Bellevue, the cradle from its birth.

Understand, we do not mean that opium is discarded, but only its abuse—always abuse in such heroic doses. One hundred grains in the hands of Dr. Clark, is a very different affair from the same amount in the hands of less skillful men; the vast experience of the one, his powers of analysis, his quick and accurate observation, would teach him how to use it, and where to stop; while, in the case of others, they would only court Death, and when he *came*, would hardly know whether at the bidding of the disease, or the doctor.

"Mihi jam, non regia Roma,
Sed vacuum Tiber placet."

Chomel, a very celebrated French physician, left us a wise morsel of advice when he said, "in the practice of medicine, if you can do no good, be certain to do no harm," and this is the key-note to the practice of every sound and intelligent medical man.

Left bunch of fives straight from the shoulder; caught his potatoe-trap sharply; abundance of the ruby; and again went to grass.

Third Round. Our doctor thinks the following proposition should become an axiom: "*If indications are scrupulously met in treatment, no drug-poisoning, with its disagreeable and dangerous perturbations, will occur, however large the dose of the drug may be.*"

Violent attack, defended by the following paraphrasis: *However large the dose of the drug may be, if the symptoms of poisoning are scrupulously met, with all their disagreeable and dangerous perturbations, by giving still more of the drug*, (similia similibus curantur), *the patient will certainly "go under," or the drug is not worth one cent.*

Last round fearfully scientific; a rally; doctor thrown by a cross-buttock, and back broken; time called, no response; and sponge thrown up.

In this playful sketch, you will perceive, we have not attempted to give a history of metro-peritonitis, or the differentiation between the symptoms of this disease, and opium-poisoning, as it would take up too much time, and any text-book will supply the deficiency; but we wish to advert to our point, the different methods of dying under these two conditions, the first by asthenia, the last by apnœa. As the pulse never ran above 100 but once, and fell next day, and as no indications of sinking were at any time apparent, it would seem as if the heart was capable of holding its own; but, at the end of 44 hours, with 123 grains of opium, and six more as the *coup de grace*, and "the indications being more than met," it does seem highly probable—to a man up a tree—as if the respiratory function was about to be extinguished. And, again, as opium is a stimulant narcotic, the "therapeutical paradox" is not very clear; stimulant at first, sedative still later; then why quibble about cardiac action as contradistinguished from arterial?

That *savan* down in Indiana did no good by putting in his small paddle; it was a very "pretty quarrel as it stood," to quote Sir Lucius O'Trigger—and all in the world he effected, was to demonstrate more conclusively that the necessity *does* exist for every physician to strive to arrest such heroic practice among the people.

A very large proportion of new beginners adopt much from the various medical journals, and it is only after a very sad experience that they recognize such fallacies. Our Indiana friend had better come up, and spend *one* more session, probably he would absorb more correctly the teachings of the "Rush Fathers," and not suppose, because they enunciated a general truth, they were to be held responsible for all its perversions.

To finish, we desire to say a few words about a curious article on the "Doctors of Chicago," published a few weeks back in the Sunday Times. We say curious, because the writer, evidently with a praiseworthy desire to serve the profession, and furnish an item to his paper, has been led into gross partiality, and has done manifest injustice to many noble men. His apologetic remarks in a measure explain this away, but he should never attempt to write on any subject of which he has so limited a knowledge, nor to draw his inspirations from a weak, interested triangle, who, taking advantage of his good nature, and under the influence of several nips of "Red Eye," induce him to sing their praise, and the seeming merits of their friends. Should this gentleman ever desire to write a thorough and truthful history of the "Doctors of Chicago," we beg him to call upon us, as we are comparatively new comers, with no interests to serve, no axes to grind, and hence can give him impartial statements without guile or malice. He must first recognize the fact, that the physicians of this city are counted by the hundreds, but the learned and highly educated among them by the units; and there probably exists no city on the face of this globe, of so large a population, with so many incompetents, and so many foisted into high positions through social, church and monied influences, but whose reputations, as scientific men, are purely factitious. He knows well enough that, to become an efficient practitioner, requires a literary, classical and scientific foundation to begin on. You cannot build an enduring superstructure on quicksand, nor make a silk purse out of a sow's ear,—and without this, the majority of physicians must degenerate into mere routinists, and treat their patients mechanically, just as a carpenter would repair a table, or a tinker mend a pot. Of course they look so wise, use so many technicalities, spout so many platitudes—and the more ignorant, the more they do this—that the credulous public swallow unresistingly their doses, and form their estimate of the parties' worth from their own braggings. Why, my dear sir, you have barely touched the merits of some of the most eminent men of this country; some of vast erudition, ripe experience, and most comprehensive minds not even named; while on the other hand, you have overdrawn, and given the most florid descriptions of a certain class, recognized as distinguished only by themselves, or their adherents, and by all others as rather inflated egotists. Let us examine the record, and in a few words touch up your pictures.

First comes that "small, restless phantom in black" . . . complex prescriptions . . . . no alcohol, but lots of tinctures . . . . political harangue . . . . temperance exhortation.

Second comes the "uterine madman" . . . . dark grim looking cannon . . . . down *she* goes, . . up *it* goes, . . . . female humanity.

Third comes the "pretty man with the big feet" . . . the *soi disant* successor of Brainard (*E pluribus unum*, *vide*

N. Y. Independent) . . . . church interests . . . . cupidity . . . . public notice . . . . morbid taste . . . . . court room . . . . . arsenic . . . . tail between his legs.

With these representatives of the three great branches of our science (or art), medicine, obstetrics and surgery, we must stop, for we have not only taken too much space, but if we go on at this rate people will begin to think us ill-natured or prejudiced, when in fact, we know none of the parties, and have formed our opinions upon their publicly recognized traits.

But to still further advise the writer, or any other wishing to write up the doctors, he must again remember that they are divided into rings or cliques—the hospital cliques; the college cliques; the society cliques; and the outside cliques—each damning the other collectively and individually, and to obtain anything like accurate *data*, each must be consulted as to the merits of its various members, and then each member individually to recount his marvellous cures, incomparable remedies, and extensive experience, and about the time he has completed this augean labor, and gotten pretty well mixed up, he will find himself in that bewildered state of the rustics, as described in Goldsmith's Deserted Village, when—

> "With words of learned length, and thundering sound,
> The gaping Poliuto stood, and gazed around,
> And as he gazed, still the wonder grew,
> That DEATH warn't *kilt* by this sapient crew."

My dear Professor, it is time to bring these ramblings to a stop, and before so doing to offer some extenuation for the small amount of venom distilled from the point of our pen; but we have said to you before, that it is partly our province to attack and endeavor to correct—by censure or praise—the hollowness, the cant, the hypocrisy, and the pretensions of many medical men in these regions, and if they will not take warning, and strive to amend their ways, we shall prod them again and again, hoping that in the long run, the very sense of shame and respect for public opinion may compel a stricter observance of the rules of our code, and a kinder regard for the rights of others. The moment any man convinces the profession that he is capable, honest, and a true gentleman, none will be found to indorse him, and wish him *bonnie fortune* more earnestly than ourselves; but just let him try the other tack, and, swelling up with the consciousness of possessing a little money, or assuming an importance to which he is in no way entitled, continue to play the " tricks of the cur," we shall immediately prick the pompous wind-bag, and every time the occasion forces us to thrust, deeper and deeper shall be the wounds. With this explanation, and the last one, we make our parting bow.

" VIATOR."

CHICAGO, April, 1871.

